# Impact of decompressive laminectomy on the functional outcome of patients with metastatic spinal cord compression and neurological impairment

**DOI:** 10.1007/s10585-019-10016-z

**Published:** 2020-01-20

**Authors:** Alexander Younsi, Lennart Riemann, Moritz Scherer, Andreas Unterberg, Klaus Zweckberger

**Affiliations:** Department of Neurosurgery, University of Heidelberg, INF 400, 69120 Heidelberg, Germany

**Keywords:** Metastatic spinal cord compression (MSCC), Spinal metastases, Laminectomy, Decompressive surgery, Ambulation, Frankel grade

## Abstract

Metastatic spinal cord compression (MSCC) is a frequent phenomenon in advanced tumor diseases with often severe neurological impairments. Affected patients are often treated by decompressive laminectomy. To assess the impact of this procedure on Karnofsky Performance Index (KPI) and Frankel Grade (FG) at discharge, a single center retrospective cohort study of neurologically impaired MSCC-patients treated with decompressive laminectomy between 2004 and 2014 was performed. 101 patients (27 female/74 male; age 66.1 ± 11.5 years) were identified. Prostate was the most common primary tumor site (40%) and progressive disease was present in 74%. At admission, 80% of patients were non-ambulatory (FG A–C). Imaging revealed prevalently thoracic MSCC (78%). Emergency surgery (< 24 h) was performed in 71% and rates of complications and revision surgery were 6% and 4%, respectively. At discharge, FG had improved in 61% of cases, and 51% of patients had regained ambulation. Univariate predictors for not regaining the ability to walk were bowl dysfunction (p = 0.0015), KPI < 50% (p = 0.048) and FG < C (p = 0.001) prior to surgery. In conclusion, decompressive laminectomy showed beneficial effects on the functional outcome at discharge. A good neurological status prior to surgery was key predictor for a good functional outcome.

## Introduction

Spinal metastases are a common manifestation of malignant diseases and have been reported in autopsy-studies in 30–70% of cancer patients since the 1950s [1–3]. Due to improvements in diagnostic and treatment of cancer, along with an aging population, the number of patients surviving years beyond their cancer diagnosis has increased and consequently also the incidence of spinal metastases [4–6]. Breast, prostate, lung and kidney tumors most commonly disseminate into the spine [7]. Metastases are thereby most frequently located within the thoracic spine, followed by the lumbar and cervical spine [7, 8]. In more than 30% of cases, spinal metastases are discontinuously located on multiple vertebral-levels [9, 10].

Despite local back-pain being the initial symptom in most patients, spinal metastases are frequently diagnosed not before neurological deficits occur [9, 11, 12]. These may include sensory and motor disturbances as well as autonomic dysfunction [11, 13]. Progression of the epidural masses leads to metastatic spinal cord compression (MSCC) and might finally result in complete and irreversible paraplegia, unless timely treatment is initiated [14]. This most serious and devastating sequel of spinal metastases is termed malignant epidural spinal cord compression (MESCC) and occurs in 3–5% of all cancer patients [15, 16]. Although MESCC does not directly alter life expectancy, its’ severe clinical course results in rapid deterioration of neurological function culminating in a paraplegic status. Finally, this loss of ambulation leads to a significant reduction of the patients’ quality of life [7, 11]. It is understood that MESCC has to be treated as an oncological emergency, requiring rapid decision-making if neurological function should be preserved [13, 17]. In this context, early therapeutic intervention as well as a good neurological status prior to treatment-initiation are repeatedly accounted for a better functional outcome [18–20].

Treatment options for MSCC include the administration of corticosteroids, chemotherapy, different forms of radiotherapy as well as different surgical approaches [6, 17, 21]. Surgery, however, remains the only treatment option leading to immediate relief of neural compression. In addition, it can ascertain histopathological diagnosis [17]. Indications widely accepted for decompression surgery include rapid neurologic deterioration, pain unresponsive to conservative treatment or radio-resistant tumors [22]. Decompressive laminectomy has been the surgical treatment of choice for MSCC patients, lowering mortality and morbidity rates [15], but several reports on inadequate decompression and poor neurological outcome have initiated a critical discussion about the use of this technique [9, 23–28]. Apart from that, individualized surgical approaches were further developed [29–31] and despite the fact that the presence of spinal metastases makes most subsequent therapies palliative, radical surgical approaches encompassing gross total tumor resection with replacement of vertebral bodies combined with anterior or posterior stabilization were established in order to offer further treatment alternatives aiming for oncological cure [32–35]. Nevertheless, indication for surgery has to take into account that patients with spinal metastases often suffer from multiple disseminated metastases and severe comorbidities, and thus mostly are in a reduced general condition with limited life expectancy [19, 36, 37]. Considering these issues, radical and curative tumor resection often appears challenging when surgery should not impair the patients’ remaining quality of life [38].

Although several studies have evaluated prognostic factors that may affect survival [39–41] or the psychological status of MSCC patients, only limited information is available on their quality of life before and after treatment [42–46]. Especially in cancer patients, quality of life is strongly dependent on the ambulatory status which in turn is mostly affected by MSCC. Independent from comorbidities and tumor expansion, decompressive laminectomy remains a straightforward surgical technique that might have the potential to improve neurological function in selected MSCC patients, potentially preventing loss of ambulation and improving quality of life.

The aim of the current study therefore was to present data on the early postoperative ambulatory status of neurologically impaired MSCC patients without spinal instability who were surgically treated by decompressive laminectomy and to identify factors that may reinstitute their ability to walk.

## Methods

### Patient selection

**Fig. 1:**
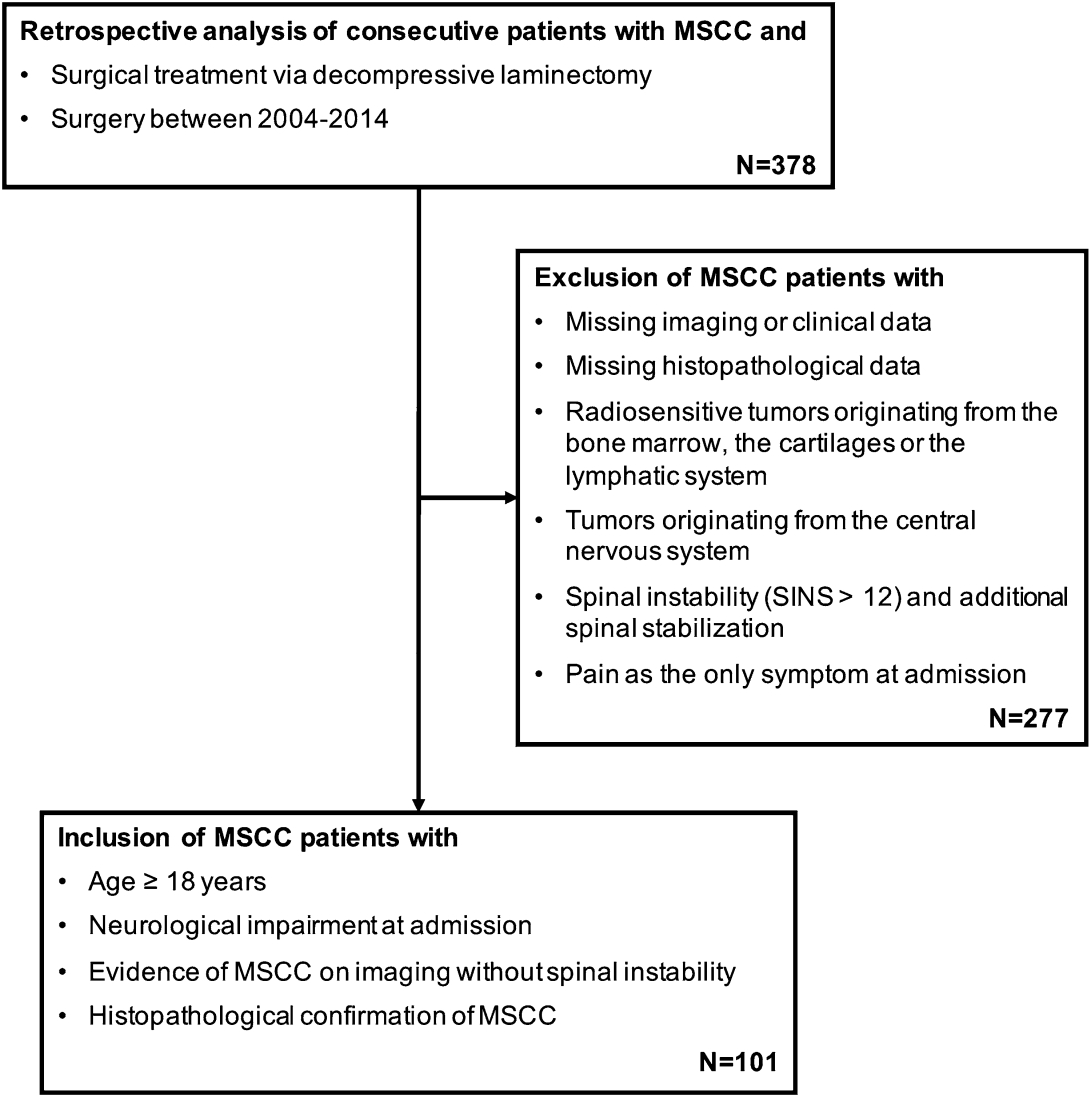
Flow-diagram of patient selection (*MSCC* metastatic spinal cord compression, *SINS* spinal instability neoplastic score)

A single center retrospective analysis of all consecutive patients with metastatic spinal cord compression who underwent decompressive laminectomy with the primary goal of maximum posterior decompression at our institution between 2004 and 2014 was performed. Adult patients (≥ 18 years) with neurological impairment at admission, a tissue-proven diagnosis of solid primary tumor and evidence of MSCC by an epidural mass on imaging were further analyzed. Patients with pain as their only symptom at admission as well as radiosensitive tumors originating from the bone marrow, the cartilages or the lymphatic system and tumors originating from the central nervous system were excluded. Furthermore, cases in which spinal instability according to the Spinal Instability Neoplastic Score (SINS > 12) was present and in which additional stabilization of the vertebral column was required were excluded as well (Fig. 1). The local standing committee of ethnical practice approved the protocol of this study.

### Clinical evaluation and outcome assessment

Information was collected from the patients’ hospital records including demographics, clinical presentation and duration of symptoms, preoperative imaging findings, surgical details, perioperative management and surgical or non-surgical complications as well as the pre- and postoperative neurological status. Perioperative mortality was defined as death during the in-hospital stay.

For morphological evaluation of MSCC, the 6-point Epidural Spinal Cord Compression (ESCC) scale [47] was determined as a consensus decision of three independent raters on preoperative imaging [47]. To determine spinal stability, the SINS score [48], which assesses tumor-related instability by adding together scores for spinal location, pain, lesion bone quality, radiographic alignment, vertebral body collapse and posterolateral involvement of the spinal elements was calculated for every patient [49]. Furthermore, the modified Tokuhashi score [39] was determined for each patient. This score uses six parameters (general condition, extraspinal bone metastases, metastases in the vertebral body, metastases to major organs, primary tumor site, spinal cord palsy) ranging from 0 to 5 points with a total score of 15 points and can be used for pretreatment evaluation of metastatic spinal tumor prognosis [39]. Karnofsky performance status (KPS) scale [50] and Frankel Grade (FG) [51] at admission and at the day of discharge, obtained by the treating physicians were collected to assess the patients’ functional outcome. The ambulatory status at discharge was thereby used as the primary outcome parameter and ambulation was defined as a Frankel Grade of D or E.

### Statistical analysis

For statistical comparison, subgroups of patients with and without an ambulatory status at admission as well as at discharge were formed. The p-values for categorical variables (gender, primary (first) symptom, ambulation, imaging, location of metastases, complications, revisions, etc.) were calculated with Fisher’s exact test. For comparison of continuous variables (age, inpatient stay, number of metastases, time from onset to surgery, ESCC, Tokuhashi score, KPS, FG, strength level, duration of paresis, time point of surgery, etc.), a two-sided Student’s t test was used. Additionally, associations between the described variables and the retrieval of ambulation at the time of discharge were assessed in univariate analysis. No adjustment for multiple testing was performed as this was an exploratory analysis. All statistical analyses were conducted using GraphPad Prism 7.0b. A p-value < 0.05 was considered statistically significant.

## Results

### Patient demographics

A total of 101 eligible patients (74 male, 27 female) with a mean age of 66.1 ± 11.5 years (mean ± SD) was identified. Spinal metastases originated from the prostate in 40 (40%), the lung in 23 (23%), and the breast in 11 (11%) of cases. Other tumors (including kidney, melanoma, larynx, and GI.) accounted for 19 (19%) of the metastases. Most patients (74%) were in a progressive stage of the underlying malignant disease with at least one additional, extraspinal metastasis. In eight patients (8%), the existence of a malignant disease had still been unknown at the time of presentation. (Table 1).

**Table 1:** Patient demographics and imaging findings

Characteristics	Values
Number of patients	101
Sex (%)	
Male	74 (73%)
Female	27 (27%)
Age (median, IQR / mean ± mean)	66, 57–75 years / 66.1 ± 11.5 years
Origin of metastases (%)	
Prostate	40 (40%)
Lung	23 (22%)
Breast	11 (11%)
Others	19 (19%)
Unknown	8 (8%)
Progressive disease (%)^a^	75 (74%)
Number of metastases (%)	
Solitary	31 (31%)
2–5	40 (40%)
> 5	30 (29%)
Location of metastases (%)	
Cervical	7 (7%)
Thoracic	79 (78%)
Lumbar	15 (15%)
Sacral	0 (0%)
Rad. sign of myelopathy (%)	38 (38%)
ESCC scale (%)	
1a	1 (1%)
1b	0 (0%)
1c	2 (2%)
2	26 (26%)
3	72 (71%)
SINS score (%)	
SINS 0–6	82 (81%)
SINS 7–12	19 (19%)
SINS 13–18	0 (0%)

*IQR* inter quartile range, *SD* standard deviation, *ESCC* epidural spinal cord compression, *SINS* spinal instability neoplastic score

^a^At least one additional extraspinal metastasis

### Imaging

MR images of the spine were performed in 93 patients (92%). Since contraindications for MR imaging, the remaining 8% of patients received CT scans only. Thirty-one patients (31%) had a single metastasis in only one vertebral body, whereas 70 patients (69%) presented with multiple lesions, sometimes located in distant parts of the spinal column. Most metastases involved the thoracic spine (n = 79, 78%), whereby the spinal level Th 4–7 were affected in a majority of cases (43%), followed by the lumbar (n = 15, 15%) and the cervical spine (n = 7, 7%). The cervico-thoracic or thoraco-lumbar junctions were affected in 3 (3%) and 1 (1%) case, respectively. Morphological evaluation of MSCC revealed an ESCC grade of 1a in 1 (1%), of 1c in 2 (2%), of 2 in 26 (26%) and of 3 in 72 (71%) patients. No patient had an ESCC grade of 0, or 1b. Spinal stability measured by the SINS score showed complete stable conditions in 81% of cases (n = 82) and an average SINS score of 5 ± 2.26 (mean ± SD). Intermediate stability was present in 19 patients (19%) and no patient had an instable spine. (Table 1).

### Clinical presentation

The most relevant symptoms determined by the patients prior to admission and mostly the reason for patient referral to our institution were motor palsy in 63% of cases (n = 64), followed by pain in 20% (n = 20) and sensory deficits in only 12% (n = 12) of cases. These symptoms had been present since a median of 5 days prior to hospitalization (IQR 2–14 days).

Neurological examination at admission revealed paresis in 101 patients (100%) with muscle strength of grade 3 or less according to the British Medical Research Council (BMRC) grading system [52] and thus, the inability to move the corresponding extremities against gravity. Sensory deficits were present in 83 patients (82%) and abnormal urinary sphincter function was present in 60 patients (60%) whereas bowel dysfunction only occurred in 25 patients (25%). Nearly half of the patients suffered from back pain (n = 49, 49%) while radiating pain was rare (n = 13, 13%). Most importantly, all patients (100%) showed impaired ambulation (FG A–D) and 81 patients (80%) had even completely lost ambulation at admission (FG A–C). Nearly all patients (96%) thus were unable to work or carry out normal activities of daily living measured by the Karnofsky Performance Index (KPI score < 80%). (Tables 2 and 3).

**Table 2:** Comparison of preoperative non-ambulatory (n = 81) and ambulatory (n = 20) patients

Characteristic	All	Non-ambulatory preoperatively	Ambulatory preoperatively	p-value
Number of patients	101	81	20	
First symptom (%)				
Paresis	64 (63%)	56 (69%)	8 (40%)	0.0205^2^
Pain	20 (20%)	12 (15%)	8 (40%)	0.0240^2^
Sensory deficit	12 (12%)	9 (11%)	3 (15%)	0.7006^2^
Bowl/bladder dysfunction	5 (5%)	4 (5%)	1 (5%)	> 0.9999^2^
Duration of first symptom (median, IQR / mean ± SD)	5, 2–14 days / 17.3 ± 42.5 days	5, 2–14 days / 10.9 ± 16.1 days	10, 2–35 days / 42.85 ± 86.8 days	0.0022^1^
KPI (median, IQR / mean ± SD)	40, 30–50% / 42.8 ± 13.4%	40, 30–40% / 38.3 ± 8.5%	60, 50–70% / 61 ± 14.5%	< 0.0001^1^
FG on admission (%)				
Grade A	17 (17%)	17 (21%)	0 (0%)	0.0209^2^
Grade B	11 (11%)	11 (14%)	0 (0%)	0.1151^2^
Grade C	53 (52%)	53 (65%)	0 (0%)	< 0.0001^2^
Grade D	20 (20%)	0 (0%)	20 (100%)	< 0.0001^2^
Grade E	0 (0%)	0 (0%)	0 (0%)	
Symptoms on admission (%)				
Paresis	101 (100%)	81 (100%)	20 (100%)	> 0.9999^2^
Back pain	49 (49%)	36 (44%)	13 (65%)	0.1346^2^
Radiating pain	13 (13%)	6 (7%)	7 (35%)	0.0035^2^
Sensory deficit	83 (82%)	68 (84%)	15 (75%)	0.3434^2^
Bladder dysfunction	60 (59%)	54 (67%)	6 (30%)	0.0045^2^
Bowl dysfunction	25 (25%)	25 (31%)	0 (0%)	0.0027^2^
Duration of paresis (median, IQR / mean ± SD)	4.5, 2–10 days / 11.6 ± 26.3 days	4, 1.5–7 days / 7.2 ± 11.5 days	10, 3–21 days / 30.1 ± 52.5 days	0.0005^1^
Degree of paresis (%)				< 0.0001^2^
> Grade 3/5 BMRC	24 (24%)	7 (9%)	17 (85%)	
< Grade 4/5 BMRC	77 (76%)	74 (91%)	3 (15%)	
Location of metastases (%)				
Cervical	6 (6%)	4 (5%)	2 (10%)	0.3396^2^
Thoracic	81 (80%)	66 (82%)	15 (75%)	0.5374^2^
Lumbar	13 (13%)	10 (12%)	3 (15%)	0.7177^2^
Sacral	0 (0%)	0 (0%)	0 (0%)	
Rad. sign of myelopathy (%)	38 (38%)	33 (41%)	5 (25%)	0.3024^2^
ESCC scale (%)				
1a	1 (1%)	0 (0%)	1 (5%)	0.1980^2^
1c	2 (2%)	0 (0%)	2 (10%)	0.0376^2^
2	26 (26%)	19 (23%)	7 (35%)	0.3912^2^
3	72 (71%)	62 (77%)	10 (50%)	0.0272^2^
Tokuhashi score (%)				
0–8	63 (62%)	55 (68%)	8 (40%)	0.0372^2^
9–11	35 (35%)	26 (32%)	9 (45%)	0.3025^2^
12–15	3 (3%)	0 (0%)	3 (15%)	0.0068^2^
ASA score (%)				
< ASA 3	26 (26%)	14 (18%)	8 (40%)	0.0681^2^
> ASA 2	75 (74%)	63 (82%)	12 (60%)	
Time to surgery after adm (median, IQR / mean ± SD)	13, 8–25 h / 24 ± 36 h	12, 8–23.5 h / 23 ± 38 h	22, 10–48 h / 31 ± 26 h	0.4067^1^
Levels decompressed (median, IQR / mean ± SD)	2, 1–2 / 1.9 ± 0.9	2, 1–2 / 1.8 ± 0.8	2, 1–2.7 / 1.9 ± 0.9	0.8482^1^
Duration of surgery (median, IQR / mean ± SD)	130, 105–160 min /138.4 ± 49.5 min	130, 105–150 min / 135 ± 46 min	142, 97.7–200 min / 151 ± 60 min	0.4074^1^
Complications (%)	6 (6%)	6 (7%)	0 (0%)	0.3508^2^
Revision surgery (%)	4 (4%)	4 (5%)	0 (0%)	0.5821^2^
Hospital length of stay	8, 6–12 days	8, 5.5–12 days	9, 7–13 days	0.1897^1^
(median, IQR/mean ± mean)	9 ± 4.7 days	9 ± 5 days	10 ± 5 days	
FG on discharge (%)				
Grade A	9 (9%)	9 (11%)	0 (0%)	0.1978^2^
Grade B	3 (3%)	3 (4%)	0 (0%)	> 0.9999^2^
Grade C	27 (27%)	27 (34%)	0 (0%)	0.0013^2^
Grade D	49 (49%)	38 (47%)	11 (55%)	0.2823^2^
Grade E	12 (12%)	3 (4%)	9 (45%)	0.011^2^

*SD* standard deviation, *IQR* inter quartile range, *FG* Frankel grade, *KPI* Karnofsky performance index, *ESCC* epidural spinal cord compression, *SINS* Spinal instability neoplastic score, *ASA* American Society of Anesthesiologists

^1^Student’s t-test

^2^Fishers exact test

**Table 3:** Comparison of postoperative non-ambulatory (n = 40) and ambulatory (n = 61) patients

Characteristic	All	Non-ambulatory postoperatively	Ambulatory postoperatively	p-value
Number of patients	101	40	61	
Sex (%)				< 0.0001^2^
Male	74 (73%)	27 (67.5%)	47 (77%)	
Female	27 (27%)	13 (32.5%)	14 (23%)	
First symptom (%)				
Paresis	64 (63%)	30 (75%)	34 (67%)	0.4895^2^
Pain	20 (20%)	5 (13%)	15 (24%)	0.2016^2^
Sensory deficit	12 (12%)	4 (10%)	8 (13%)	0.7592^2^
Bowl/bladder dysfunction	5 (5%)	1 (2.5%)	4 (7%)	0.7092^2^
Duration of first symptom (median, IQR / mean ± SD)	5, 2–14 days / 17.3 ± 42.5 days	7, 2–14 days / 12.85 ± 18.5 days	5, 2.5–14 days / 20.1 ± 52.6 days	0.3229^1^
KPI on admission				
< 50%	73 (72%)	38 (95%)	35 (57%)	< 0.0001^2^
> 40%	28 (28%)	2 (5%)	26 (43%)	
FG on admission (%)				
Grade < D	81 (80%)	40 (100%)	41 (67%)	< 0.0001^2^
Grade > C	20 (20%)	0 (0%)	20 (33%)	
Symptoms on admission (%)				
Paresis	101 (100%)	81 (100%)	20 (100%)	> 0.9999^2^
Back pain	49 (49%)	17 (43%)	32 (52%)	0.4164^2^
Radiating pain	13 (13%)	2 (5%)	11 (18%)	0.0711^2^
Sensory deficit	83 (82%)	32 (80%)	51 (84%)	0.7911^2^
Bladder dysfunction	60 (59%)	29 (73%)	31 (51%)	0.0387^2^
Bowl dysfunction	25 (25%)	19 (48%)	6 (10%)	< 0.0001^2^
Duration of paresis (median, IQR / mean ± SD)	4.5, 2–10 days / 11.6 ± 26.3 days	4, 1–14 days / 9.3 ± 15.3 days	5, 2–10 days / 13.1 ± 31.6 days	0.4268^1^
Degree of paresis (%)				
> Grade 3/5 BMRC	24 (24%)	1 (3%)	23 (62%)	< 0.0001^2^
< Grade 4/5 BMRC	77 (76%)	39 (97%)	38 (38%)	
Ambulatory status on admission				
Able to walk	20 (20%)	0 (0%)	20 (33%)	< 0.0001^2^
Unable to walk	81 (80%)	40 (100%)	41 (67%)	
Duration of inability to walk (median, IQR / mean ± SD)	48, 24–96 h / 71 ± 76.5 h	24, 24–72 h / 70 ± 89.7 h	48, 24–96 h / 72 ± 62.9 h	0.9107^2^
Tokuhashi score (%)				
0–8	63 (62%)	30 (75%)	33 (54%)	0.0382^2^
9–11	35 (35%)	10 (25%)	25 (41%)	0.1347^2^
12–15	3 (3%)	0 (0%)	3 (5%)	0.2752^2^
ASA score (%)				
< ASA 3	26 (26%)	5 (14%)	17 (28%)	0.1337^2^
> ASA 2	75 (74%)	32 (86%)	43 (72%)	
Time to surgery (%)				
< 24 h after admission	72 (72%)	30 (75%)	42 (70%)	0.6537^2^
> 24 h after admission	28 (28%)	10 (25%)	18 (30%)	
Duration of surgery (median, IQR / mean ± mean)	130, 105–160 min / 138.4 ± 49.5 min	130, 105–153 min / 139 ± 50 min	131, 100–161 min / 138 ± 49.7 min	0.9277^1^
Complications (%)	6 (6%)	3 (7.5%)	3 (5%)	0.2589^2^
Revision surgery (%)	4 (4%)	1 (3%)	3 (5%)	> 0.9999^2^
Hospital length of stay (median, IQR / mean ± SD)	8, 6–12 days / 9 ± 4.7 days	7.5, 4–12 days / 8.5 ± 4.8 days	8, 6–12 days / 9.4 ± 4.7 days	0.3676^1^
Improvement on discharge (%)				
KPI	75 (74%)	24 (60%)	51 (84%)	0.0107^2^
Frankel Grade	61 (61%)	11 (28%)	50 (82%)	< 0.0001^2^
Ambulation	41 (41%)	0 (0%)	41 (67%)	< 0.0001^2^
Paresis	69 (68%)	23 (58%)	46 (75%)	0.0802^2^
Back pain	23 (23%)	7 (50%)	16 (50%)	> 0.9999^2^
Radiating pain	7 (7%)	0 (0%)	7 (64%)	0.1923^2^
Sensory deficit	15 (15%)	3 (10%)	12 (25%)	0.1454^2^
Bladder dysfunction	19 (19%)	4 (14%)	15 (54%)	0.0041^2^
Bowl dysfunction	5 (5%)	2 (11%)	3 (60%)	0.0482^2^

*SD* standard deviation, *IQR* inter quartile range, *FG* Frankel Grade, *KPI* Karnofsky Performance Index, *BMRC* British Medical Research Council, *ESCC* Epidural Spinal Cord Compression, *SINS* Spinal Instability Neoplastic Score, *ASA* American Society of Anesthesiologists

^1^Students t-test

^2^Fishers exact test

### Surgical management and complications

Following informed consent, surgical treatment was performed as an emergency procedure within 24 h after admission in 72 cases (71%). The overall median time to surgery was 13 h (IQR 8–24.75 h) after admission, and 65 h (IQR 32.5–100 h) after loss of ambulation. Due to the vast progression of tumor disease, patients showed severe systemic co-morbidities with an ASA score (American Society of Anesthesiologists Physical Status Classification System score) of III in 62% (n = 60) and IV in 15% (n = 15) of cases. Intraoperatively, a median of 2 spinal segments (IQR 1–2) were posteriorly decompressed by laminectomy.

Surgery-related complications occurred in four patients (4%), consisting of three cases of secondary hemorrhage which all required revision surgery and one case of wound infection which required revision surgery as well. Additionally, general complications occurred in two patients (2%), both displaying symptoms of cardiorespiratory insufficiency. One of those two patients developed a myocardial infarction and died during the in-hospital stay. Overall complication rate was therefore 6%, revision rate 4% and mortality rate 1%. Patients could be discharged from the surgical ward after 9 ± 4.7 days (mean ± SD) (Tables 2 and 3).

### Postoperative outcome and impact on ambulation

At discharge, 83 patients (84%) reported that their symptoms had overall improved. Especially palsies showed good recovery (improvement in 73% of cases) followed by alleviation of pain (radiating pain in 54% and back pain in 47% of cases) whereas sensory deficits as well as bladder or bowl dysfunction were often persistent (improvement in 18%, 24%, and 20% of cases, respectively).

**Fig. 2:**
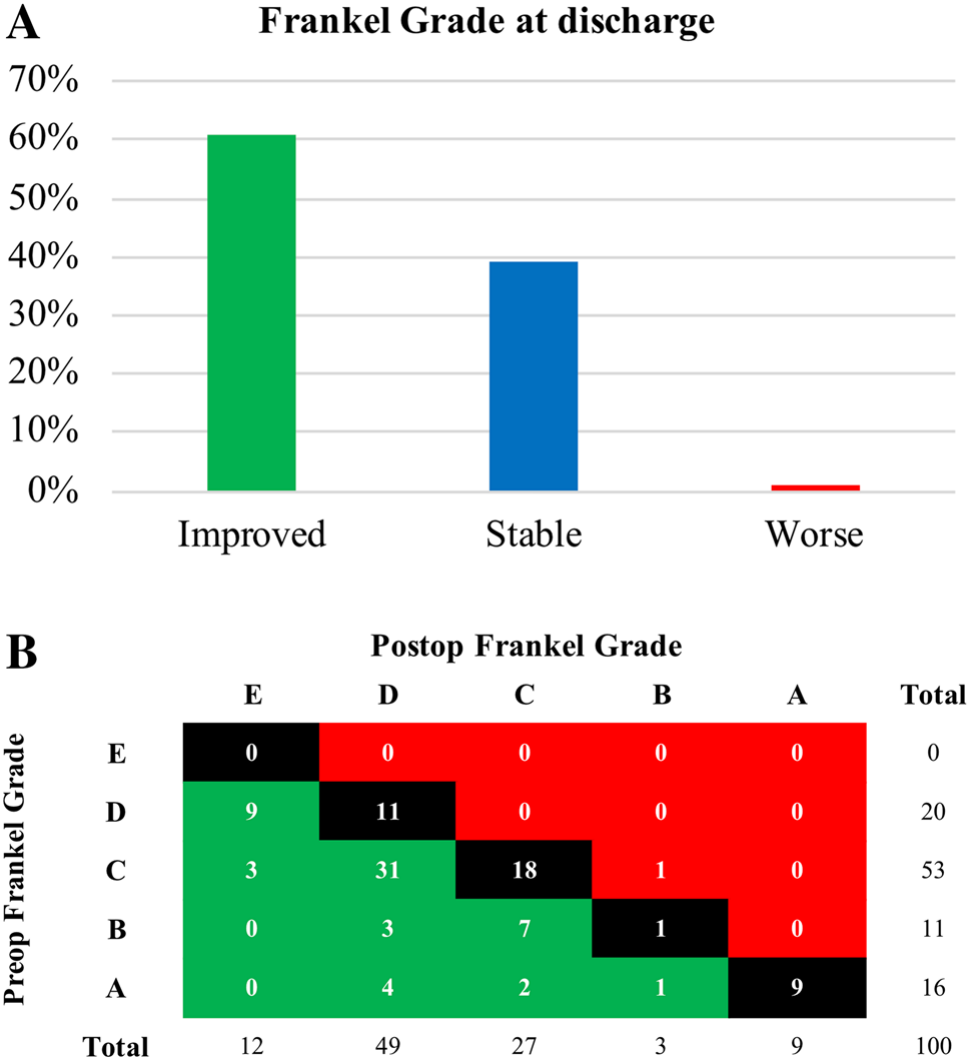
**a** Number of patients with either improved, stable or worsened Frankle Grade at discharge. **b** Differences between preoperative Frankel Grade and Frankel Grade on discharge (postoperative)

Pre-operatively impaired neurological function (Frankel Grade A–D) had improved by ≥ 1 grade in the Frankel Grade in 61% of patients at discharge (Fig. 2a). To emphasize, 25% of all severely impaired patients (Frankel Grade A and B prior to surgery) and 51% of all non-ambulatory patients (Frankel Grade A–C) had regained ambulation after surgery (Fig. 2b). Overall, 61 patients (61%) were ambulatory at discharge (Frankel Grade D and E) compared to 20 patients (20%) prior to surgery.

Functional improvement in the KPI score was observed in 75 patients (75%) and at discharge, 27% of patients had a KPI score ≥ 80 compared to 4% prior to surgery (Tables 2, 3, 4).

**Table 4:** Univariate analysis of factors associated with regaining the ability to walk after surgery for 81 non-ambulatory patients

	Ambulation regained		Relative risk (95% CI)	p-value
	yes (n = 41) (%)	no (n = 40) (%)		
Demographic factors				
Age > 70 years	55	45	1.171 [0.693–2.000]	0.6528
Female sex	38	62	0.600 [0.280–1.258]	0.2122
Primary tumor unknown	71	29	1.087 [0.939–1.296]	0.264
Primary tumor				
Prostate	52	48	1.041 [0.600–1.1814]	> 0.9999
Lung	48	52	0.887 [0.428–1.830]	0.8036
Breast	50	50	0.976 [0.283–3.364]	> 0.9999
Progressive disease	48	52	0.884 [0.673–1.143]	0.441
Clinical presentation:				
First symptom				
Pain	58	42	1.366 [0.496–3.1812]	0.756
Sensory deficit	56	44	1.220 [0.379–3.962]	> 0.9999
Paresis	46	54	0.845 [0.618–1.137]	0.3374
Bladder/bowl disorder	75	25	2.927 [0.439–20.020]	0.6156
Duration of first symptom > 7 d	38	62	1.25 [0.919–1.744]	0.2324
Symptoms on admission				
Back pain	53	47	0.818 [0.518–1.305]	0.4736
Radiating pain	67	33	1.951 [0.442–8.797]	0.6755
Sensory deficit	53	47	1.098 [0.898–1.370]	0.3793
Paresis	50	50	1 [0.914–1.096]	> 0.9999
Bladder disorder	54	46	0.82 [0.591–1.116]	0.238
Bowl disorder	24	76	0.3 [0.134–0.640]	0.0015
Duration of paresis > 7 days	33	67	1.22 [0.963–1.597]	0.115
Paresis < 4/5 BMRC grade	47	53	5.854 [0.988–36.28]	0.1088
Non-ambulatory since > 48 h	62	38	0.749 [0.508–1.079]	0.1689
KPI < 50%	46	54	4.39 [1.162–17.4]	0.048
Frankel grade				
A	24	76	0.300 [0.109–0.788]	0.0148
B	27	73	0.366 [0.110–1.172]	0.1157
C	64	34	1.746 [1.257–2.567]	0.001
Frankel grade < C	25	75	0.325 [0.154–0.649]	0.001
Imaging and clinical course				
Number of metastases				
Solitary	44	56	0.767 [0.397–1.463]	0.4769
2–5	62	38	1.576 [0.936–2.735]	0.1161
> 5	41	59	0.675 [0.3261.373]	0.3256
Location of metastases				
Cervical spine	40	60	0.65 [0.135–3.109]	0.6755
Thoracic spine	54	46	1.128 [0.934–1.403]	0.2258
Lumbar spine	56	44	1.22 [0.379–3.962]	0.9999
ESCC scale (%)				
2	47	53	0.878 [0.404–1.899]	0.7976
3	52	48	1.041 [0.808–1.351]	0.7976
Radiological signs of myelopathy	48	52	0.918 [0.541–1.552]	> 0.9999
SINS score				
0–6	48	52	0.918 [0.728–1.143]	0.5609
7–12	60	40	1.463 [0.596–3.66]	0.569
Tokuhashi score				
0–8	45	55	0.813 [0.587–1.101]	0.2351
9–11	62	38	1.561 [0.824–3.034]	0.2351
Emergency operation in > 24 h	55	45	0.944 [0.722–1.226]	0.7976
Operation in > 48 h	43	57	1.03 [0.881–1.122]	0.7123
ASA score > 2	49	51	1.665 [0.645–4.415]	0.3822
Duration of surgery > 120 min	51	49	1.025 [0.660–1.598]	> 0.9999
Complications occured	50	50	0.585 [0.162–2.08]	> 0.9999
Revision surgery necessary	75	25	2.927 [0.439–20.02]	0.6156
Hospital stay > 7 days	40	60	0.650 [0.297–1.386]	0.3116

The Fisher’s exact test was used for univariate analysis

*BMRC* British Medical Research Council, *KPI* Karnofsky performance index, *ASA* American Society of Anesthesiologists, *CI* confidence interval

### Comparison of preoperative ambulatory and non-ambulatory patients

Statistical analysis of 81 ambulatory (Frankel Grade D–E) and 20 non-ambulatory (Frankel Grade A–C) patients prior to surgery revealed significant differences in perioperative variables (Table 3): Non-ambulatory patients more frequently had paresis as their first symptom (p < 0.05), whereas preoperative ambulatory patients more commonly were suffering from pain (p < 0.05). Furthermore, the median KPI was lower for non-ambulatory patients compared to ambulatory patients (p < 0.01). At admission, radiating pain was more common in ambulatory patients (p < 0.01) whereas non-ambulatory patients experienced bladder and bowl dysfunction more frequently (both p < 0.01). While all patients suffered from motor palsy when admitted to our institution, its’ duration was shorter but its’ degree higher (p < 0.01 and p < 0.001 respectively) in non-ambulatory patients. Non-ambulatory patients more often showed spinal cord compression with no visible CSF (ESCC scale = 3) in imaging studies (p < 0.05) and had a predicted survival period of less than 6 months (p < 0.05) according to the modified Tokuhashi score (0–8). In return, ambulatory patients more frequently had a predicted survival period of 1 year or more (Tokuhashi score 12–15; p < 0.01). While the time to surgery was shorter in non-ambulatory patients, no significant difference could be noted (p = 0.06). Nevertheless, more non-ambulatory patients received surgical treatment within 48 h after admission (p < 0.05). No further variables were found to be significantly different between both groups (Table 2).

### Identification of factors affecting postoperative ambulation

In univariate analyses, male sex, a better neurological status prior to surgery (for Frankel Grade and KPI), the absence of bladder or bowl dysfunction as well as a lower degree of motor palsy and a lower Tokuhashi score were associated with an ambulatory status at the time of discharge. No other factors were significantly correlated with the ability to walk after surgery (Table 3).

### Identification of predictors for regaining the ability to walk at discharge

Statistical analyses of a subgroup of 81 patients who had lost the ability to walk prior to surgery showed significant negative associations with regaining ambulation at discharge for the following variables: Presence of bowl dysfunction at admission (RR 0.3; 95% CI 0.134–0.640; p = 0.0015), KPI < 50% prior to surgery (RR 4.39; 95% CI 1.162–17.4; p = 0.048) and Frankel Grade < C prior to surgery (RR 0.325; 95% CI 0.154–0.649; p = 0.001). Of note, patients who regained ambulation at discharge had presented with a median duration of their first symptom of 4 days (IQR 2.5–10.5 days) compared to 6.5 days (IQR 2–14) in patients who remained non-ambulatory and a median duration of muscle weakness of 3 days (IQR 2–7 days) compared to 4 days (IQR 1–13.5 days). These differences, however, did not reach statistical significance. No further clinical, imaging, surgical or pathological parameter was significantly affecting the recovery of ambulation at discharge (Table 4).

## Discussion

In this study of 101 neurologically impaired MSCC-patients without spinal instability that received decompressive laminectomy, 74% showed improved motor function and 51% had regained the ability to walk at discharge while overall complication rate as well as revision and mortality rates (6%, 4%, and 1%, respectively) were low. In univariate analyses, absence of bowl dysfunction, better neurological status as well as smaller surgery in terms of decompressed spinal levels were associated with postoperative retrieval of the ability to walk.

It is noteworthy that in contrast to many other published series [53, 54], all MSCC patients in our study had impaired motor function and 80% were unable to walk prior to surgery. To our knowledge, our study is the only clinical series that solely focusses on the surgical treatment of neurologically impaired MSCC patients. Additionally, our study population was older (66.1 ± 11.52 years mean ± SD) and had a more extensive metastatic disease (74% with extraspinal metastasis) than many of the MSCC patient cohorts in the literature [55]. Furthermore, all MSCC patients that were treated by decompressive laminectomy in our study had a SINS score between 0 and 12, and therefore no relevant spinal instability. It needs to be emphasized that MSCC patients who underwent other surgical procedures (e.g. posterolateral fusion), which are mostly required when spinal instability is present, were excluded in our current study. Our findings hence should only be applied to MSCC patients with neurological impairment, a SINS score ≤ 12 and an extensive metastatic disease with limited life expectancy.

### Differences in characteristics of preoperative ambulatory and non-ambulatory patients

Loss of ambulation due to MSCC is mainly caused by motor palsy and spinal ataxia. Back pain or radiating pain may limit the patients´ mobility to some extent as well, but the objective Frankel Grade we used to assess the ambulatory status of MSCC patients does not inquire these symptoms. Our findings reflect the often-rapid progression of MSCC into MESCC which makes affected patients an oncological emergency [13, 16]. As expected, the KPI was lower in non-ambulatory patients, since it is influenced by the patients’ ability to walk.

Further imaging analyses revealed a trend towards thoracic localization of spinal metastases in non-ambulatory patients with a higher rate of radiological signs of myelopathy which might be affected by the anatomical narrowing of the spinal canal in this region. Pretreatment evaluation of prognosis by the modified Tokuhashi score predicted a shorter survival period for non-ambulatory patients. However, it must not be forgotten that this score itself already includes KPI and Frankel Grade as two of its six prognostic factors. In addition, due to recent improvements in specific cancer therapies, and hence increased survival time of some MSCC patients, the modified Tokuhashi score, in which the primary tumor constitutes a major factor in estimating life expectancy, is thought to be increasingly limited [39, 56].

Non-ambulatory MSCC patients have been described to require more extensive surgery in terms of decompressed vertebral levels and to incur more complications [18]. Due to possible difficulties in decompressing the spine in these cases, it has been recommended to perform early surgical interventions before MSCC patients become non-ambulatory [34, 35, 57, 58]. In our study, there were no statistically significant differences in the extent or duration of surgery as well as the length of hospital stay between preoperative ambulatory and non-ambulatory patients. However, complications and revision surgeries only occurred in non-ambulatory patients which might be influenced by their worse overall health status, assessed by preoperative ASA scores. Likewise, time to surgery was shorter for non-ambulatory patients. In contrast to other studies, these findings did not reach statistical significance in our analysis. The indication to perform early surgery on ambulatory MSCC patients without neurological impairment in order to prevent surgical complications should therefore be critically discussed [18].

### Decompressive laminectomy to maintain or regain ambulatory ability

In their recent multicenter randomized study, Patchell et al. compared radiotherapy alone with both surgery and radiotherapy and revealed that aggressive surgical decompression and instrumented stabilization had half the mortality rate compared to radiotherapy alone. Additionally, patients in the surgical arm retained the ability to walk for significantly longer than those in the radiotherapy arm without spending increased time in the hospital [59]. Although the study has been critically discussed due to a possible selection bias towards better outcome in the surgical arm as well as poor functional results after radiotherapy alone when compared with the literature [60], it confirmed the importance of surgery in the treatment of MSCC patients.

Today, extensive surgical techniques to treat MSCC patients with e.g. circumferential instrumentation and fusion or corporectomy and cage graft placement from an anterolateral, posterolateral or retroperitoneal approach are available [61]. It has to be noted that goals of surgery with such approaches usually go beyond restoration or preservation of neurological function and include deformity correction and stabilization as well as oncologic control [62].

However, rates of complications for the surgical treatment of MSCC patients reported in the literature with more extensive approaches are high and range between 10 and 48% [54, 55, 63–68]. Our current data reinforces this problem: MSCC patients were of higher age, had progressive disease in most of cases, a reduced functional status (KPI) prior to surgery and severe systemic symptoms (ASA 3 or 4). These are some of the typically increased risk factors for such local and systemic complications after surgery [55]. Laminectomy, a surgical technique that allows fast decompression of the spinal cord in cases of MSCC with the possibility of obtaining a histological sample or further tumor debulking has been pushed into an increasingly marginal role in the last decades [69]. Although surgical complication rates are generally low, the technique has fallen into disrepute for causing vertebral collapse and possible neurologic deterioration which in return may have resulted in the increased use of radiotherapy for MSCC treatment in the past [7]. Nevertheless, our data suggests that decompressive laminectomy might provide significant outcome benefits for a specific cohort of MSCC patients. In our study, all patients had a SINS score < 13, and therefore no evidence for spinal instability. Because the SINS score was specifically developed to assess the stability of the spine in MSCC patients, it has been proven to be reliable and reproducible with a sensitivity and specificity for potentially unstable lesions of 95.7% and 79.5% respectively [49]. In addition, 98% of the patients in our series had an ESCC scale of 2 or 3 and therefore profound spinal cord compression, 100% suffered from motor weakness at admission and 80% were unable to walk prior to surgery since only 24–96 h.

Compared to other surgical series in the literature, the postoperative impact of decompressive laminectomy on the ambulatory status of our MSCC patients was high: Chong et al. reported an improved Frankel Grade in 20% of 105 MSCC patients after single-stage posterior decompression and stabilization with a complication rate of 10% and a revision rate of 10% [64]. Fourney et al. published a series of 72 MSCC patients treated by transthoracic vertebrectomy which lead to functional improvement in 59% of cases with a complication rate of 35% and 3% mortality [35]. Jansson et al. assessed 282 MSCC patients who underwent different surgical approaches, reporting functional improvement in 70% of cases with a complication rate of 20% and 13% mortality in the first months after surgery [55]. In our study, no MSCC patient lost the ability to walk after surgery, 74% had functional improvement at discharge and 51% had regained the ability to walk while overall complication rate as well as revision and mortality rates (4%, 2% and 1% respectively) were low. Even completely paraplegic patients became walkers at discharge after emergency decompressive laminectomy in 25% of cases.

Like other authors, we found that a better neurological status (KPI > 40%, FG > C) prior to surgery is associated with the ability to walk at discharge [34, 35, 70, 71]. Moreover, our data suggests that higher KPI (> 40%) and better FG (> C) at admission are predictors even for non-ambulatory patients to regain the ability to walk after surgery. Surprisingly, duration of motor weakness or duration of the inability to walk prior to surgery had no significant impact on the ambulatory status at discharge, although trends towards shorter durations could be observed. Likewise, an earlier timepoint of surgery after admission of MSCC patients (</> 24 h) showed no association with postoperative ambulation. We assume, that these findings might be related to the small sample size in our study. Nevertheless, in order to alleviate damage to the spinal cord and thus allow for better recovery of neurological function, prompt surgical intervention should be performed in MSCC patients before edema, venous congestion and secondary vascular injury due to compression occur [18, 59].

In our analyses, a lower modified Tokuhashi score (0–8) as well as the presence of bladder- and bowl dysfunction at admission were associated with the inability to walk at discharge. Moreover, the presence of bowl dysfunction was a predictor for non-ambulatory patients to remain unable to walk after surgery. Although the Tokuhashi score itself is partly determined by the patients’ ambulatory status, we deem it a useful tool to predict not only prognosis for survival but also for postoperative ambulation. Interestingly, Tokuhashi et al. already recommend conservative treatment for MSCC patients with a total score of 8 or less due to a predicted survival period of < 6 months [39]. To this recommendation, our data adds the finding that these patients may also have a worse functional outcome when treated surgically. The presence of bowl dysfunction at admission might be an additional prognostic factor to predict the postoperative functional outcome of MSCC patients.

### Limitations

Our study is primarily limited by its retrospective design and the corresponding lack of a prospective follow up assessing the long-term neurological status, development of spinal instability and the survival of MSCC patients. Moreover, we are unable to present data on further adjuvant treatments. Although we demonstrate objective and immediate effects of decompressive laminectomy on the ambulatory status, the alteration of ambulation over time which is expected to decrease depending on e.g. local radiation or local tumor recurrence therefore remains unknown. Similarly, possible secondary instability in e.g. patients with laminectomy over the cervico-thoracic or thoraco-lumbar junction cannot be addressed. However, information on direct effects of the surgical treatment on the functional status are equally important for affected patients and treating physicians. Secondly, due to its single center design and its relatively long time period, our study is prone to selection bias and heterogeneity in treatment due to secular changes. Nevertheless, decompressive laminectomy as a surgical technique did not change during the 10-year period of our analysis and there was no significant difference in surgery time or rate of complications between patients who were operated within the first 5 vs. the last 5 years of the study. Thirdly, the onset of motor symptoms, usually reported by the patients themselves, is only loosely defined in our series, which limits our results regarding neurologic improvement and outcome after surgery. Prospective studies are certainly needed to provide better data on the long-term effect of decompressive laminectomy and to guide clinical decision-making in the surgical treatment of MSCC patients.

## Conclusion

Our data demonstrates a beneficial effect of decompressive laminectomy on the ambulatory status at discharge in the treatment of 101 neurologically impaired MSCC patients: 61 (61%) patients could walk at discharge compared to only 20 (20%) who were able to ambulate preoperatively. More importantly, patients with preserved sensation only or even complete loss of any motor or sensory function (FG A + B) regained ambulation in 25% of cases. Additionally, surgical (4%) and general complications (2%) as well as mortality (1%) after decompressive laminectomy were low. In univariate analysis, the absence of bowl dysfunction as well as a better neurological status prior to surgery were associated with postoperative retrieval of the ability to walk.

## References

[1] Abrams HL, Spiro R, Goldstein N (1950). Metastases in carcinoma; analysis of 1000 autopsied cases. Cancer.

[2] Fornasier VL, Horne JG (1975). Metastases to the vertebral column. Cancer.

[3] Ortiz Gómez JA (1995). The incidence of vertebral body metastases. Int Orthop.

[4] Aziz NM (2002). Cancer survivorship research: challenge and opportunity. J Nutr.

[5] Aziz N, Rowland J, Society AC, Ries LAG, Smith MA, Gurney JG (2003). Trends and advances in cancer survivorship research: challenge and opportunity. Semin Radiat Oncol.

[6] Jacobs WB, Perrin RG (2001). Evaluation and treatment of spinal metastases: an overview. Neurosurg Focus.

[7] Ibrahim A, Crockard A, Antonietti P, Boriani S, Bünger C, Gasbarrini A (2008). Does spinal surgery improve the quality of life for those with extradural (spinal) osseous metastases? An international multicenter prospective observational study of 223 patients. J Neurosurg Spine.

[8] Hirabayashi H, Ebara S, Kinoshita T, Yuzawa Y, Nakamura I, Takahashi J (2003). Clinical outcome and survival after palliative surgery for spinal metastases: palliative surgery in spinal metastases. Cancer.

[9] Gilbert RW, Kim JH, Posner JB (1978). Epidural spinal cord compression from metastatic tumor: diagnosis and treatment. Ann Neurol.

[10] O’Rourke T, George CB, Redmond J, Davidson H, Cornett P, Fill WL (1986). Spinal computed tomography and computed tomographic metrizamide myelography in the early diagnosis of metastatic disease. J Clin Oncol.

[11] Levack P, Graham J, Collie D, Grant R, Kidd J, Kunkler I (2002). Don’t wait for a sensory level–listen to the symptoms: a prospective audit of the delays in diagnosis of malignant cord compression. Clin Oncol (R Coll Radiol).

[12] Onimus M, Papin P, Gangloff S (1996). Results of surgical treatment of spinal thoracic and lumbar metastases. Eur Spine J.

[13] McCurdy MT, Shanholtz CB (2012). Oncologic emergencies. Crit Care Med.

[14] Botterell EH, Fitzgerald GW (1959). Spinal cord compression produced by extradural malignant tumours; early recognition, treatment and results. Can Med Assoc J.

[15] Witham TF, Khavkin YA, Gallia GL, Wolinsky J-P, Gokaslan ZL (2006). Surgery insight: current management of epidural spinal cord compression from metastatic spine disease. Nat Clin Pract Neurol.

[16] Mak KS, Lee LK, Mak RH, Wang S, Pile-Spellman J, Abrahm JL (2011). Incidence and treatment patterns in hospitalizations for malignant spinal cord compression in the United States, 1998–2006. Int J Radiat Oncol Biol Phys.

[17] Prasad D, Schiff D (2005). Malignant spinal-cord compression. Lancet Oncol.

[18] Chaichana KL, Woodworth GF, Sciubba DM, McGirt MJ, Witham TJ, Bydon A (2008). Predictors of ambulatory function after decompressive surgery for metastatic epidural spinal cord compression. Neurosurgery.

[19] Ribas ESC, Schiff D (2012). Spinal cord compression. Curr Treat Options Neurol.

[20] Fan Y, Zhou X, Wang H, Jiang P, Cai S, Zhang J (2016). The timing of surgical intervention in the treatment of complete motor paralysis in patients with spinal metastasis. Eur Spine J.

[21] Al-Qurainy R, Collis E (2016). Metastatic spinal cord compression: diagnosis and management. BMJ.

[22] Gerszten PC, Welch WC (2000). Current surgical management of metastatic spinal disease. Oncology (Williston Park).

[23] Sorensen S, Borgesen SE, Rohde K, Rasmusson B, Bach F, Boge-Rasmussen T (1990). Metastatic epidural spinal cord compression. Results of treatment and survival. Cancer.

[24] Black P (1979). Spinal metastasis: current status and recommended guidelines for management. Neurosurgery.

[25] Findlay GF (1984). Adverse effects of the management of malignant spinal cord compression. J Neurol Neurosurg Psychiatry.

[26] Constans JP, de Divitiis E, Donzelli R, Spaziante R, Meder JF, Haye C (1983). Spinal metastases with neurological manifestations. Review of 600 cases. J Neurosurg.

[27] Hall AJ, Mackay NN (1973). The results of laminectomy for compression of the cord or cauda equina by extradural malignant tumour. J Bone Joint Surg Br.

[28] Klimo PJ, Thompson CJ, Kestle JRW, Schmidt MH (2005). A meta-analysis of surgery versus conventional radiotherapy for the treatment of metastatic spinal epidural disease. Neuro Oncol.

[29] Harrington KD (1984). Anterior cord decompression and spinal stabilization for patients with metastatic lesions of the spine. J Neurosurg.

[30] Cooper PR, Errico TJ, Martin R, Crawford B, DiBartolo T (1993). A systematic approach to spinal reconstruction after anterior decompression for neoplastic disease of the thoracic and lumbar spine. Neurosurgery.

[31] Sundaresan N, Digiacinto GV, Hughes JE, Cafferty M, Vallejo A (1991). Treatment of neoplastic spinal cord compression: results of a prospective study. Neurosurgery.

[32] Sundaresan N, Steinberger AA, Moore F, Sachdev VP, Krol G, Hough L (1996). Indications and results of combined anterior. Posterior approaches for spine tumor surgery. J Neurosurg.

[33] Xu R, Garcés-Ambrossi GL, McGirt MJ, Witham TF, Wolinsky J-P, Bydon A (2009). Thoracic vertebrectomy and spinal reconstruction via anterior, posterior, or combined approaches: clinical outcomes in 91 consecutive patients with metastatic spinal tumors. J Neurosurg Spine.

[34] Gokaslan ZL, York JE, Walsh GL, McCutcheon IE, Lang FF, Putnam JB (1998). Transthoracic vertebrectomy for metastatic spinal tumors. J Neurosurg.

[35] Fourney DR, Abi-Said D, Rhines LD, Walsh GL, Lang FF, McCutcheon IE (2001). Simultaneous anterior-posterior approach to the thoracic and lumbar spine for the radical resection of tumors followed by reconstruction and stabilization. J Neurosurg.

[36] Yao KC, Boriani S, Gokaslan ZL, Sundaresan N (2003). En bloc spondylectomy for spinal metastases: a review of techniques. Neurosurg Focus.

[37] Cho D-C, Sung J-K (2009). Palliative surgery for metastatic thoracic and lumbar tumors using posterolateral transpedicular approach with posterior instrumentation. Surg Neurol.

[38] Klimo P, Dailey AT, Fessler RG (2004). Posterior surgical approaches and outcomes in metastatic spine-disease. Neurosurg Clin N Am.

[39] Tokuhashi Y, Matsuzaki H, Oda H, Oshima M, Ryu J (2005). A revised scoring system for preoperative evaluation of metastatic spine tumor prognosis. Spine.

[40] Tomita K, Kawahara N, Kobayashi T, Yoshida A, Murakami H, Akamaru T (2001). Surgical strategy for spinal metastases. Spine.

[41] Tokuhashi Y, Matsuzaki H, Toriyama S, Kawano H, Ohsaka S (1990). Scoring system for the preoperative evaluation of metastatic spine tumor prognosis. Spine (Phila Pa 1976).

[42] Tang Y, Qu J, Wu J, Liu H, Chu T, Xiao J (2016). Effect of surgery on quality of life of patients with spinal metastasis from non-small-cell lung cancer. J Bone Joint Surg Am.

[43] Petteys RJ, Spitz SM, Rory Goodwin C, Abu-Bonsrah N, Bydon A, Witham TF (2016). Factors associated with improved survival following surgery for renal cell carcinoma spinal metastases. Neurosurg Focus.

[44] Lei M, Liu Y, Tang C, Yang S, Liu S, Zhou S (2015). Prediction of survival prognosis after surgery in patients with symptomatic metastatic spinal cord compression from non-small cell lung cancer. BMC Cancer.

[45] Harris M (2016). Quality of life in patients with malignant spinal cord compression: a review of evidence-based literature. Int J Palliat Nurs.

[46] Goodwin CR, Schoenfeld AJ, Abu-Bonsrah NA, Garzon-Muvdi T, Sankey EW, Harris MB (2016). Reliability of a spinal metastasis prognostic score to model 1-year survival. Spine J.

[47] Bilsky MH, Laufer I, Fourney DR, Groff M, Schmidt MH, Varga PP (2010). Reliability analysis of the epidural spinal cord compression scale. J Neurosurg Spine.

[48] Fourney DR, Frangou EM, Ryken TC, Dipaola CP, Shaffrey CI, Berven SH (2011). Spinal instability neoplastic score: an analysis of reliability and validity from the spine oncology study group. J Clin Oncol.

[49] Fisher CG, DiPaola CP, Ryken TC, Bilsky MH, Shaffrey CI, Berven SH (2010). A novel classification system for spinal instability in neoplastic disease: an evidence-based approach and expert consensus from the Spine Oncology Study Group. Spine (Phila Pa 1976).

[50] Karnofsky DA, Abelmann WH, Craver LF, Burchenal JH (1948). The use of the nitrogen mustards in the palliative treatment of carcinoma. With particular reference to bronchogenic carcinoma. Cancer.

[51] Frankel HL (1969). Ascending cord lesion in the early stages following spinal injury. Paraplegia.

[52] Paternostro-Sluga T, Grim-Stieger M, Posch M, Schuhfried O, Vacariu G, Mittermaier C (2008). Reliability and validity of the Medical Research Council (MRC) scale and a modified scale for testing muscle strength in patients with radial palsy. J Rehabil Med.

[53] Rompe JD, Hopf CG, Eysel P (1999). Outcome after palliative posterior surgery for metastatic disease of the spine–evaluation of 106 consecutive patients after decompression and stabilisation with the Cotrel-Dubousset instrumentation. Arch Orthop Trauma Surg.

[54] Wise JJ, Fischgrund JS, Herkowitz HN, Montgomery D, Kurz LT (1999). Complication, survival rates, and risk factors of surgery for metastatic disease of the spine. Spine (Phila Pa 1976).

[55] Jansson K-A, Bauer HCF (2006). Survival, complications and outcome in 282 patients operated for neurological deficit due to thoracic or lumbar spinal metastases. Eur Spine J.

[56] Morgen SS, Lund-Andersen C, Larsen CF, Engelholm SA, Dahl B (2013). Prognosis in patients with symptomatic metastatic spinal cord compression: survival in different cancer diagnosis in a cohort of 2321 patients. Spine (Phila Pa 1976).

[57] Sciubba DM, Gokaslan ZL, Suk I, Suki D, Maldaun MVC, McCutcheon IE (2007). Positive and negative prognostic variables for patients undergoing spine surgery for metastatic breast disease. Eur Spine J.

[58] Ogihara S, Seichi A, Hozumi T, Oka H, Ieki R, Nakamura K (2006). Prognostic factors for patients with spinal metastases from lung cancer. Spine (Phila Pa 1976).

[59] Patchell RA, Tibbs PA, Regine WF, Payne R, Saris S, Kryscio RJ (2005). Direct decompressive surgical resection in the treatment of spinal cord compression caused by metastatic cancer: a randomised trial. Lancet.

[60] Rades D, Abrahm JL (2010). The role of radiotherapy for metastatic epidural spinal cord compression. Nat Rev Clin Oncol.

[61] Kaloostian PE, Yurter A, Zadnik PL, Sciubba DM, Gokaslan ZL (2014). Current paradigms for metastatic spinal disease: an evidence-based review. Ann Surg Oncol.

[62] Chen LH, Chen WJ, Niu CC, Shih CH (2000). Anterior reconstructive spinal surgery with Zielke instrumentation for metastatic malignancies of the spine. Arch Orthop Trauma Surg.

[63] Lau D, Leach MR, Than KD, Ziewacz J, La Marca F, Park P (2013). Independent predictors of complication following surgery for spinal metastasis. Eur Spine J.

[64] Chong S, Shin S-H, Yoo H, Lee SH, Kim K-J, Jahng T-A (2012). Single-stage posterior decompression and stabilization for metastasis of the thoracic spine: prognostic factors for functional outcome and patients’ survival. Spine J.

[65] Sundaresan N, Sachdev VP, Holland JF, Moore F, Sung M, Paciucci PA (1995). Surgical treatment of spinal cord compression from epidural metastasis. J Clin Oncol.

[66] Finkelstein JA, Zaveri G, Wai E, Vidmar M, Kreder H, Chow E (2003). A population-based study of surgery for spinal metastases. Survival rates and complications. J Bone Joint Surg Br.

[67] Choi D, Fox Z, Albert T, Arts M, Balabaud L, Bunger C (2015). Prediction of quality of life and survival after surgery for symptomatic spinal metastases: a multicenter cohort study to determine suitability for surgical treatment. Neurosurgery.

[68] Turner I, Minhas Z, Kennedy J, Morris S, Crockard A, Choi D (2015). Cost of surgery for symptomatic spinal metastases in the United Kingdom. World Neurosurg.

[69] Lee C-H, Kwon J-W, Lee J, Hyun S-J, Kim K-J, Jahng T-A (2014). Direct decompressive surgery followed by radiotherapy versus radiotherapy alone for metastatic epidural spinal cord compression: a meta-analysis. Spine (Phila Pa 1976).

[70] Gokaslan ZL, Aladag MA, Ellerhorst JA (2000). Melanoma metastatic to the spine: a review of 133 cases. Melanoma Res.

[71] Jackson RJ, Loh SC, Gokaslan ZL (2001). Metastatic renal cell carcinoma of the spine: surgical treatment and results. J Neurosurg.

